# A Role for Global DNA Methylation Level and *IL2* Expression in the Transition From Acute to Chronic Low Back Pain

**DOI:** 10.3389/fpain.2021.744148

**Published:** 2021-09-27

**Authors:** Olivia C. Eller, Nicole Glidden, Brittany Knight, Noelle McKearney, Mallory Perry, Katherine M. Bernier Carney, Angela Starkweather, Erin E. Young, Kyle M. Baumbauer

**Affiliations:** ^1^Department of Anatomy and Cell Biology, University of Kansas Medical Center, Kansas City, KS, United States; ^2^Department of Genetics and Genome Sciences, UConn Health, Farmington, CT, United States; ^3^Center for Advancement in Managing Pain, School of Nursing, University of Connecticut, Storrs, CT, United States; ^4^Department of Neuroscience, UConn Health, Farmington, CT, United States; ^5^Department of Anesthesiology, University of Kansas Medical Center, Kansas City, KS, United States

**Keywords:** low back pain, epigenetics, cytokines, hypersensitivity, chronic pain

## Abstract

**Objectives:** The transition from acute low back pain (aLBP) to chronic LBP (cLBP) results from a variety of factors, including epigenetic modifications of DNA. The aim of this study was to (1) compare global DNA (gDNA) methylation and histone acetylation at LBP onset between the aLBP and cLBP participants, (2) compare mRNA expression of genes with known roles in the transduction, maintenance, and/or modulation of pain between the aLBP and cLBP participants, (3) compare somatosensory function and pain ratings in our participants, and (4) determine if the aforementioned measurements were associated.

**Methods:** A total of 220 participants were recruited for this prospective observational study following recent onset of an episode of LBP. We retained 45 individuals whose gDNA was of sufficient quality for analysis. The final sample included 14 participants whose pain resolved within 6 weeks of onset (aLBP),15 participants that reported pain for 6 months (cLBP), and 16 healthy controls. Participants were subjected to quantitative sensory testing (QST), blood was drawn via venipuncture, gDNA isolated, and global DNA methylation and histone acetylation, as well as mRNA expression of 84 candidate genes, were measured.

**Results:** Individuals that develop cLBP display multimodal somatosensory hypersensitivity relative to aLBP participants. cLBP participants also had significantly lower global DNA methylation, which was negatively correlated with interleukin-2 (*IL2)* mRNA expression.

**Discussion:** cLBP is characterized by somatosensory hypersensitivity, lower global DNA methylation, and higher *IL2* expression level compared to those whose pain will resolve quickly (aLBP). These results suggest potential diagnostic and therapeutic relevance for global DNA methylation and *IL2* expression in the pathology underlying the transition from acute to chronic LBP.

## Introduction

Low back pain (LBP) is a global health concern that affects nearly 1 in 10 people worldwide and ranks highest in terms of disability (1). Over 85% of individuals that seek care for LBP have pain in the absence of a specific underlying condition (2, 3), making it difficult to treat. Although most individuals with LBP will experience a resolution of their pain within 4–6 weeks (acute low back pain; aLBP), around 20% of individuals will develop chronic low back pain (chronic low back pain; cLBP), which lasts beyond 12 weeks and negatively impacts normal activities and quality of life (4). Variables to predict if an individual will transition from aLBP to cLBP have yet to be established, however, it can likely be attributed to a combination of genetic, epigenetic, and environmental factors (5). Current therapies for chronic pain are not universally successful, likely due to complex interactions of these factors. Therefore, establishing the mechanisms underlying the transition from aLBP to cLBP, and developing novel and individualized therapies for cLBP are extremely important.

Our previous findings (6) as well as studies from other groups (7, 8), have demonstrated associations between genetic variation and the susceptibility to chronic pain. Most commonly, these variations have been in the form of single nucleotide polymorphisms (SNPs), but other types of variations in the linear DNA sequence, e.g., copy number variations, have also been implicated in individual differences in pain and analgesia (9–11). While polymorphisms change the genetic code, individual differences can also occur in the 3 dimensional structure of the genome through epigenetic modifications. Variations in the epigenome change the physical structure of the genome through processes including DNA methylation and histone modifications, both of which affect the ease with which the linear DNA sequence can be transcribed. DNA methylation occurs when a methyl group is added to the fifth carbon of a cytosine that is adjacent to a guanine, referred to as a CpG site (12). CpG islands, or DNA sequences with a high percentage of CpG sites, are usually found within the promotor region of a gene sequence (13), which make it possible for methylation to interfere with gene transcription, generally by decreasing gene expression (12). Chronic pain patients have been found to exhibit significant differences in DNA methylation patterns (14–19). In support of this, neuropathic pain symptoms were significantly correlated with higher methylation in the CpG island of the *TRPA1* gene as well as lower *TRPA1* mRNA expression (17, 20). It has even been suggested that alterations in DNA methylation serve as a “genomic memory of pain” and can influence long-term regulation of gene expression (21). Individuals with chronic pain also display differences in histone acetylation (13, 22), which occurs on the n-terminal of the histone tail and prevents chromatin from becoming compact. This makes it more accessible for transcription factors to bind (23) typically resulting in increased transcription (24, 25). A disruption in the appropriate balance of histone acetylation and deacetylation has been implicated in the etiology of neurological disorders, including pain (26). Finally, altered gene expression is also seen in preclinical models of neuropathic pain, where at least 10% of the transcriptome becomes dysregulated (22). Given that epigenetic modifications regulate gene expression, this link further points to a potential role of epigenetics in the development of chronic painful conditions.

In the present study, we measured global DNA methylation and H4 histone acetylation, as well as mRNA expression of 84 candidate genes with known roles in the transduction, maintenance, and/or modulation of pain in peripheral blood from individuals with new onset LBP at time of recruitment (<4 weeks duration). LBP status was tracked over a 6 month period, and only participants whose pain resolved within the 1st month (aLBP) or for whom pain was still present at 6 months (cLBP) were compared to healthy controls in the present analyses. Somatosensory functioning was assessed in all participants using multiple quantitative sensory testing (QST) measurements. We hypothesized that during the acute pain phase, individuals that eventually transition to cLBP would display differences in epigenetic markers, candidate gene expression levels, and somatosensory function (QST measures) compared to those whose pain would resolve (aLBP) and healthy controls. Indeed, we found participants that would go on to develop cLBP differed in global DNA methylation status, candidate gene expression, and somatosensory function at the time of pain onset compared to aLBP participants. Identifying factors predictive of the transition to cLBP, can shed light on novel precision pain medicine interventions targeting specific pathological processes early on in order to prevent the transition all together.

## Materials and Methods

### Participants

Men and women between the ages of 18–50 years of age with recent onset of non-specific LBP who could read and write in English were invited to participate. The criteria for the LBP episode was pain anywhere in the region of the low back bound superiorly by the thoraco-lumbar junction and inferiorly by the lumbo-sacral junction, which had been present for >24 h but <4 weeks duration and was preceded by at least 1 pain free month. Recruitment took place at two urban university health systems from 2014 to 2018 following approval from the Institution Review Board.

Advertisements at primary healthcare clinics, college campuses, and in the general community were used to recruit participants. All participants provided written consent prior to study participation. We previously reported preliminary reports for baseline demographic, psychological, and somatosensory measures as well as mRNA expression of candidate genes in a subsample of the participants presented here (6, 27, 28).

Exclusion criteria for low back pain patients included: pain at another site or associated with a prior medical diagnosis of a painful condition (e.g., degenerative disc disease, herniated lumbar disc, fibromyalgia, neuropathy, rheumatoid arthritis, sciatica), previous spinal surgery, presence of neurological deficits, history of comorbidities that affect sensorimotor function (e.g., multiple sclerosis, spinal cord injury), pregnant or within 3-months postpartum, taking opioid or anticonvulsant medication, and history of diagnosed psychological disorders (e.g., bipolar disorder, schizophrenia). Eligibility for the healthy no-pain control group (for normalization of gene expression data only) included men and women (a) between 18 and 50 years of age; (b) capable of reading and writing in English; (c) with no known medical, psychological diagnoses or prescribed medications; (d) not pregnant or breastfeeding; and, (e) no recent history of pain at any location.

Participants who continued to have pain ≥ 2 on the numeric rating scale were followed up every 6 weeks until either their pain had resolved or until the end of the study (24 weeks). Participants whose pain had resolved as indicated by a McGill Pain Questionnaire Present Pain Intensity rating ≤ 1 by the 6-week time point were classified as aLBP and participants whose pain had not resolved (pain ≥ 2) at the 6-month visit were classified as cLBP.

### Procedures

After obtaining informed consent, participants were scheduled to undergo baseline data collection as soon as possible but no longer than 1 week from the time of consent. Data collection took place in a private research suite; participants completed questions about age, gender, socioeconomic status, education, lifestyle behaviors (smoking, exercise), comorbidities, and past episodes of LBP. Following completion of the questionnaires, participants underwent venipuncture for collection of blood samples and quantitative sensory testing (QST). The sequence of data collection was followed for all patients. Whole blood was collected by venipuncture into one 5-mL EDTA vacutainer and one 10-mL Paxgene blood RNA tube (PreAnalytix, Qiagen USA), labeled with a unique study identification label, and transported directly to the laboratory for processing or storage.

### Pain Measures

Participants completed both the Brief Pain Inventory (BPI) and the McGill Pain Questionnaire Short Form (MPQ-SF). BPI subscales assess the severity of pain, location of pain, pain medications, amount of pain relief in the past 24 h and the past week, and the impact of pain on daily functions. While there are instances where a summary score is used, investigators are encouraged to use the subscales (29). The BPI is a reliable and validated tool that has been used previously in LBP patients, and is sensitive to change over time (30). The MPQ-SF is a reliable self-report measure of pain perception made up of 15 verbal descriptors of sensory and affective dimensions of pain scored on a 4-point scale (0-none to 3-severe) by adding the numeric value of each pain dimension (31, 32). Higher scores indicate higher levels of sensory and affective components of pain (0–45 total scores).

### Quantitative Sensory Testing

QST uses standardized stimuli to assess both nociceptive and non-nociceptive systems (33). Following instructions and response training, participants completed a confirmation trial on the non-dominant forearm to verify the participant's understanding of the procedures. QST was used to measure participant responses on the painful lumbar region as well as the dominant forearm (remote area). A standardized protocol of administration, including testing environment, conditions, and participant instructions, was strictly followed from the same protocol described in prior analyses by our group (6, 27, 28, 34).

QST measures included pain pressure threshold, mechanical detection threshold, mechanical pain threshold, mechanical pain sensitivity, dynamic mechanical allodynia, windup ratio, vibration detection threshold, cold and warm detection thresholds, and cold and heat pain threshold. Pain pressure was measured with an algometer (range from 50 to 600 kPa) attached to a Medoc Pathway System^TM^ (Ramat Yishai, Israel) to increase pressure at a rate of 30 kPa/s until the participant indicated first pain sensation. Pressure pain threshold was determined after repeating the procedure at the same site until either the two values were recorded within 20 kPa of one another or three trials were administered. The mean of the two closest values was reported. Mechanical detection and pain threshold as well as mechanical pain sensitivity were determined using a standard set of von Frey hairs (0.25–512 mN; 0.5 mm diameter tip). The final threshold is calculated as the geometric mean of 5 series of ascending and descending stimuli intensities. Windup ratio was assessed as the mean pain rating of the stimulus trains divided by the mean pain rating to a single stimuli. Dynamic mechanical allodynia was measured with a standardized brush applied five times with a single stroke and the pain rating of each stroke was recorded. Vibration detection threshold was performed using a Rydel-Seiffer tuning fork (64 Hz, 8/8 scale) placed on the skin surface. Participants were asked to report when the vibration was no longer felt, and this number is recorded.

Thermal thresholds were determined with the Medoc Pathway System^TM^. A series of thermal testing procedures were carried out: cold detection threshold, warm detection threshold, cold pain threshold, and heat pain threshold. The mean threshold temperature of 3 consecutive measurements were calculated. The temperature stimuli were ramped at 1C/second and were terminated when the participant pressed a button.

### Genomic DNA Extraction and Purification

Blood samples were stored at 4°C for a minimum of 2 h, mixed with cold 1% fetal bovine serum and centrifuged at 3,000 rpm for 15 min to isolate the buffy coat. Buffy coat was isolated using a 200 μL pipette and stored at −80°C for subsequent extraction and purification of genomic DNA (gDNA) using the QiAmp DNA Blood Mini Kit according to manufacturer's instructions (Cat# 51104, Qiagen, Germantown, MD, USA). gDNA concentration was determined by biospectrophotometer with 1.0 μL samples (Cat # 6133000908, Eppendorf, Enfield, CT, USA).

### DNA Methylation Assay

At the time of initial assessment, the gDNA cytosine methylation level for each participant was determined by using an enzyme-linked immunosorbent assay-based commercial kit (MDQ1, Imprint^®^ Methylated DNA Quantification Kit, Sigma-Aldrich). DNA at a concentration of 150 ng was diluted with 30 μL of binding buffers and incubated at 37°C for 60 min. The samples were incubated with capture and detection antibodies and absorbance was read at 450 nm according to manufacturer's instructions. Quantification of global DNA methylation was obtained by calculating the amount of methylated cytosines in the sample relative to methylation in a positive control, which was provided by the manufacturer. All samples were run in duplicate and the global methylation level was measured and averaged to produce a single value for each participant.

### Histone Acetylation Assay

At the time of baseline assessment, the global histone acetylation level for each participant was accomplished by using an enzyme-linked immunosorbent assay-based commercial kit [Histone H4 (acetyl K8) Quantification Kit (colorimetric, abcam)]. Histone extracts were first generated from each sample using the Histone Extraction Kit (abcam). Histone extracts were incubated with antibody buffer at room temperature according to the manufacturer's instructions. The samples were then incubated with detection solution for 60 min at room temperature followed by color developer and read at 450 nm according to the manufacturer's instructions. H4K8 acetylation was quantified relative to the total histone extract amount added and the standard control, which was provided by the manufacturer. All samples were run in duplicate and the global histone acetylation was determined and averaged to produce a single value for each participant.

### Gene Expression Analysis

RNA isolation was performed using the PAXgene^TM^ total RNA isolation system (Qiagen, Valencia, CA) according to the manufacturer's protocol and was reverse transcribed using RT^2^ cDNA kit (Qiagen USA). The mRNA expression of 84 genes involved in the transduction, maintenance, and modulation of pain was determined (Neuropathic & Inflammatory RT2 Profiler PCR Array; Sabio Sciences, Valencia, CA) using qPCR performed on the ABI StepOne Plus PCR machine. After an initial incubation step, 40 cycles (95°C for 15 s and 1 min at 60°C) of PCR were performed. Relative gene expression levels were quantified using the 2^−*DD*^CT method, which normalizes data of the genes of interest to the average of three housekeeping genes β-actin (*ACTB*), *GAPDH* and Beta-microtubulin (*B2M*), and expression level was determined as fold-change relative to healthy controls. Two participants were excluded from gene expression analyses for having undetermined values in their house keeping genes but were included for all other analyses for which their data sets were complete.

### Statistical Analyses

All statistical analyses were performed using SPSS version 26 (IBM, Armonk, NY). Pearson correlation was used to examine correlations between global DNA methylation or histone acetylation and gene expression of 84 genes, QST parameters, and BPI subscales. Statistical significance was accepted at *p* < 0.05 with the exception of the gene expression data, which was adjusted to *p* < 0.001 in order to correct for multiple comparisons. Stepwise linear regression analyses were used to explore the contributions of global DNA methylation or histone H4 acetylation (step 1) and our gene of interest, interleukin 2 (*IL2*; step 2), to each QST or BPI measure. Statistical significance was set at *p* < 0.05.

## Results

### Study Participant Characteristics

**Figure 1:**
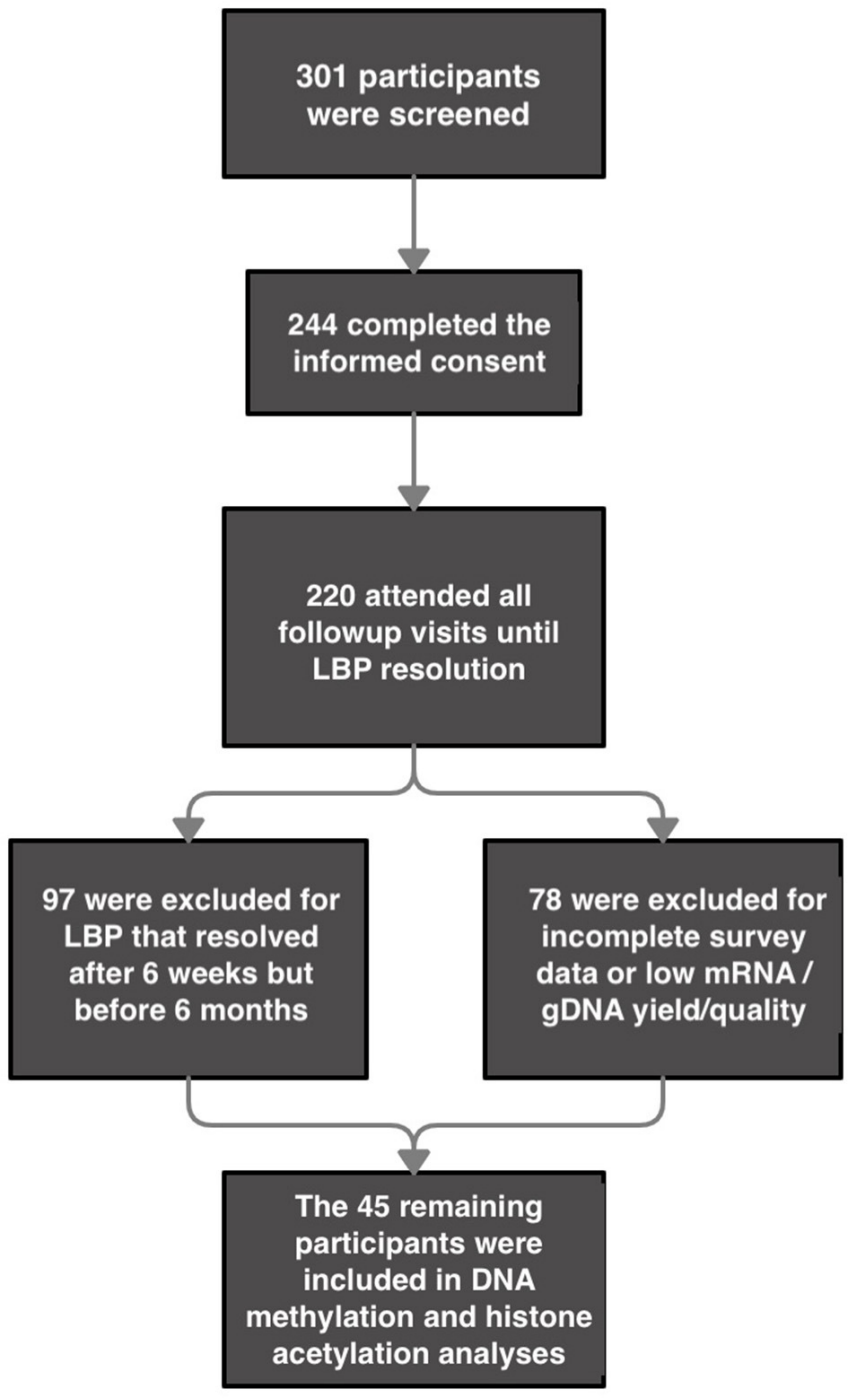
Flow Chart for participant inclusion. *2 participants were excluded from gene expression analyses for having undetermined values in their housekeeping genes but were included for all other analyses for which their data sets were complete.

A total of 220 participants were recruited for the original study; the final sample for the present analysis was comprised of 14 participants whose pain resolved within 6 weeks from onset (aLBP), 15 participants who continued to have pain for 6 months (termination of the study; cLBP), and 16 healthy controls who completed all study survey measures, QST, and whose mRNA and gDNA were of adequate quality and yield for mRNA expression analysis and the DNA methylation and histone acetylation assays, respectively (Figure 1). The remaining participants resolved at some point after 6 weeks and before 6 months and were not included in the present analysis. The results from the current sample are part of a larger funded NIH study (NCT01981382, ClinicalTrials.gov). We have previously reported differences in demographic variables and the relationship between genetic variation of the fatty acid amide hydrolase (*FAAH*) and pain sensitivity (28) as well as the catelchol-O-methyltransferase (*COMT)* and brain-derived neurotrophic factor (*BDNF)* genes and their contribution to pain chronicity within this population (6). As shown in Table 1, chi-square analysis revealed no significant differences in group distribution based on gender (females and males), age, BMI, or the percentage of smokers in each of our conditions. Our analyses did reveal that there was an over-representation of Black participants in our cLBP group, compared to what was predicted. In addition, both our cLBP and aLBP group were significantly more likely to have experienced a prior LBP episode than what was predicted following chi-square and Bonferroni correction *post-hoc* analysis for comparison to expected count.

**Table 1:** Demographic characteristics of study participants.

		**cLBP** **(*****n*** **= 15)**	**aLBP** **(*****n*** **= 14)**	**Healthy controls** **(*****n*** **= 16)**
Race	Black	11 (68.7%)^*^	3 (21.4%)	4 (25%)
	Asian	0 (0%)	3 (21.4%)	0 (0%)
	White	5 (31.3 %)	7 (50 %)	10 (62.5%)
	Other	0 (0%)	1 (7.2 %)	2 (12.5%)
Gender	Female	8 (50%)	6 (42.9%)	10 (62.5%)
	Male	8 (50%)	8 (57.1%)	6 (37.5%)
Age		39.4 (8.6)	33.5 (9.2)	36.2 (14.3)
BMI		30.5 (1.9)	27.8 (1.5)	28.6 (1.7)
Smoking	Current smoker (N, %)	8 (50%)	3 (21.4%)	3 (18.8%)
Prior LBP episodes	Yes (N, %)	13 (81.3%)^*^	10 (71.4%)^*^	-

*Data are presented as mean ± SEM or percentage.*

*Statistical analysis for categorical variables was carried out using chi-square and Bonferroni correction post-hoc analysis for comparison to expected count. Statistical analysis for age and body mass index (BMI) was carried out using ANOVA and Bonferroni post-hoc analysis. Bonferroni corrected alpha of*

* p < 0.0041 for race or ^*^p < 0.0125 for prior LBP episodes.

### Comparing Pain at the Time of Onset for aLBP vs. cLBP Participants

At the time of recruitment baseline pain thresholds were assessed using the Brief Pain Inventory (BPI) subscales. The participants that would go on to develop cLBP exhibited increased pain burden across multiple subscales compared to aLBP participants. Independent samples *t-*tests revealed that cLBP participants reported significantly higher ratings of the BPI subscales (Table 2) for Worst Pain [t(df) = −3.23, *p* < 0.05], Least Pain [t(df) = −3.39, *p* < 0.05], Average Pain [t(df) = −2.55, *p* < 0.05], Pain Now [t(df) = −3.74, *p* < 0.05], and Pain Interference [t(df) = −3.26, *p* < 0.05]. BPI data from a larger cohort of these participants has been previously published (6).

**Table 2:** Pain self-report at the time of low back pain onset by group.

	**cLBP (*n* = 15)**	**aLBP (*n* = 14)**	***p***
**BPI (scale)**			
Worst	7.0 (0.51)	4.6 (0.52)	0.003^*^
Least	4.2 (0.58)	1.8 (0.37)	0.002^*^
Average	5.5 (0.52)	3.8 (0.41)	0.016^*^
Now	5.9 (0.64)	3.1 (0.33)	0.001^*^
Relief	28.0 (5.71)	23.8 (7.72)	0.664
Interference	5.0 (0.55)	2.7 (0.44)	0.003^*^

*Data are presented as mean BPI scale rating ± SEM. BPI indicates Brief Pain Inventory.*

* *p < 0.05*.

### Somatosensory Function for aLBP vs. cLBP Participants

At the time of recruitment, QST assessments were conducted on the site of pain (low back) as well as on the non-dominant forearm as a control site. Univariate analysis of variance (ANOVA) revealed cLBP participants were more sensitive in a number of somatosensory function domains measured at the site of injury [i.e., mechanical pain sensitivity (MPS), dynamic mechanical allodynia (ALL), windup ratio at the first measurement (WUR1), and vibration detection threshold (VDT)] but also showed a higher warm detection threshold (WDT) relative to aLBP participants and healthy controls [all *F*s>3.75, *p* < 0.05 (Table 3)]. Analysis of QST measurements taken on the forearm also showed that cLBP participants reported increased WUR1 and WUR10 (both *p* < 0.05) compared to healthy controls, while no differences were observed between cLBP and aLBP participants, suggesting a moderate phenotype for aLBP participants. A similar battery of QST measures from a larger cohort of participants has been previously published (6, 27).

**Table 3:** Quantitative sensory testing (QST) measures at the time of pain onset.

**QST measurement and site**	**cLBP** **(*n* = 15)**	**aLBP** **(*n* = 14)**	**Healthy controls** **(*n* = 16)**
Pain pressure threshold (KPa)			
Low back	211.2 (38.1)	252.1 (35.3)	264.6 (40.0)
Control site	196.3 (20.6)	223.6 (19.5)	183.8 (29.4)
Mechanical detection threshold (mN)			
Low back	3.4 (0.18)	3.4 (0.17)	3.2 (0.20)
Control site	3.4 (0.13)	3.1 (0.13)	3.0 (0.089)
Mechanical pain threshold (mN)			
Low back	5.8 (0.20)	6.1 (0.19)	6.3 (0.14)
Control site	6.3 (0.13)	6.3 (0.11)	6.6 (0.057)
Mechanical pain sensitivity (NRS 0-10)			
Low back^∧^	3.9 (0.76)^*^^#^	1.8 (0.44)	1.1 (0.28)
Control site	2.1 (0.61)	1.2 (0.35)	0.72 (0.22)
Dynamic mechanical allodynia (NRS 0-10)			
Low back^∧^	2.4 (0.67)^*^^#^	0.36 (0.21)	0.49 (0.21)
Control site	0.73 (0.37)	0.048 (0.048)	0.29 (0.15)
Windup ratio measurement 1			
Low back^∧^	3.9 (0.76)^*^^#^	1.8 (0.44)	1.1 (0.28)
Control site^∧^	2.4 (0.57) ^*^	1.3 (0.36)	0.72 (0.22)
Windup ratio measurement 10			
Low back	4.6 (0.70)	3.1 (0.74)	2.4 (0.65)
Control site^∧^	3.3 (0.73)^*^	1.7 (0.42)	1.1 (0.28)
Vibration detection threshold (Hz)			
Low back^∧^	0.6 (0.12)	0.9 (0.075)	0.9 (0.078)
Control site	1.0 (0)	0.95 (0.48)	1.0 (0)
Cold detection threshold (◦C)			
Low back	27.9 (0.50)	28.8 (0.24)	29.1 (0.23)
Control site	27.5 (0.70)	28.9 (0.19)	28.7 (0.24)
Cold pain threshold (◦C)			
Low back	22.7 (1.05)	17.8 (2.62)	20.2 (2.70)
Control site	19.2 (1.65)	17.2 (2.31)	19.7 (2.26)
Warm detection threshold (◦C)			
Low back^∧^	36.3 (0.49)^*^^#^	35.0 (0.22)	34.7 (0.15)
Control site	36.5 (0.78)	35.8 (0.50)	35.0 (0.33)
Heat pain threshold (◦C)			
Low back	39.9 (0.64)	39.6 (1.19)	39.6 (0.54)
Control site	41.2 (1.13)	40.6 (1.39)	40.4 (0.72)

*Data are presented as mean QST measurement ± SEM.*

∧ *p < 0.05 between subjects difference;*

* *p < 0.05 vs. healthy controls;*

# *p < 0.05 vs. aLBP; NRS, numeric rating scale*.

### Correlations of QST in cLBP Participants Over Time

**Figure 2:**
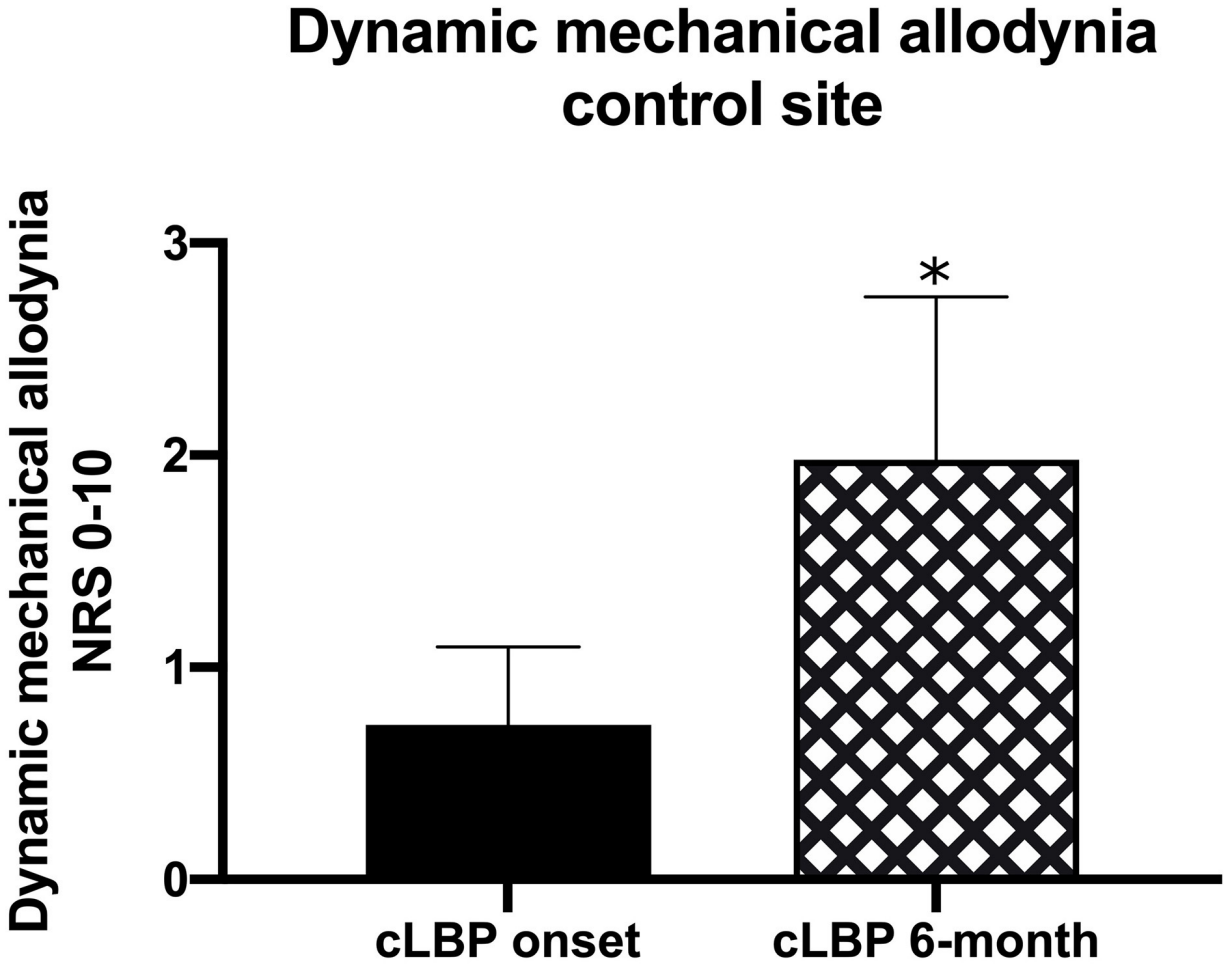
QST measurements were taken in cLBP participants at the time of LBP onset as well as 6-months into the study. We found that the dynamic mechanical allodynia (ALL) QST at the control site was significantly increased over time (^*^p < 0.05).

QST measurements were also evaluated in cLBP participants 6-months following study initiation. We used Pearson correlation to determine whether initial QST measurements were associated with measurements taken at the 6-month time point and found significant correlations in 17/24 measures (all *p* < 0.05; Table 4). Only ALL was significantly different in cLBP participants between the start of the study and the 6-month time point [*t*(df) = 2.270, *p* < 0.05; Figure 2], which increased significantly over time.

**Table 4:** Correlation of quantitative sensory testing (QST) measurements at the site of pain and the control site in cLBP participants at the time of pain onset and a 6-month follow up visit.

**QST measurement and site**	**Correlation**	***p-*** **value**	**Change from baseline (Δ)**
Pain pressure threshold			
Low back^*^	**0.644**	***p*** **= 0.007**	−67.54
Control site	0.260	*p =* 0.331	−15.74
Mechanical detection threshold			
Low back	0.105	*p =* 0.700	0.17
Control site	0.189	*p =* 0.483	−0.067
Mechanical pain threshold			
Low back^*^	**0.636**	***p*** **= 0.008**	−0.18
Control site^*^	**0.733**	***p*** **= 0.001**	−0.26
Mechanical pain sensitivity			
Low back^*^	**0.762**	***p*** **= 0.001**	−0.29
**Control site** ^*^	**0.514**	***p*** **= 0.042**	0.089
Dynamic mechanical allodynia			
Low back	0.355	*p =* 0.177	0.16
Control site^*^	**0.745**	***p*** **= 0.001**	0.82
Windup ratio measurement 1			
Low back^*^	**0.746**	***p*** **= 0.001**	−0.40
Control site^*^	**0.607**	***p*** **= 0.016**	0.18
Windup ratio measurement 10			
Low back^*^	**0.727**	***p*** **= 0.002**	0.56
Control site^*^	**0.807**	***p*** **= 0.0001**	−0.60
Vibration detection threshold			
Low back	0.435	*p =* 0.092	0.13
Control site	—+	—+	—+
Cold detection threshold			
Low back^*^	**0.530**	***p*** **= 0.035**	0.049
Control site^*^	**0.877**	***p*** **= 0.000008**	0.24
Cold pain threshold			
Low back	0.174	*p =* 0.518	0.014
Control site^*^	**0.751**	***p*** **= 0.001**	0.25
Warm detection threshold			
Low back^*^	**0.825**	***p*** **= 0.00008**	0.43
Control site^*^	**0.868**	***p*** **= 0.00001**	1.09
Heat pain threshold			
Low back^*^	**0.695**	***p*** **= 0.003**	0.34
Control site^*^	**0.644**	***p*** **= 0.007**	0.82

* *p < 0.05. + control site vibration detection threshold was the minimum score for all participants at both time points, so correlation was not calculated*.

### Global Histone H4 Acetylation Is Higher in Participants With Pain Compared to Healthy Controls

**Figure 3:**
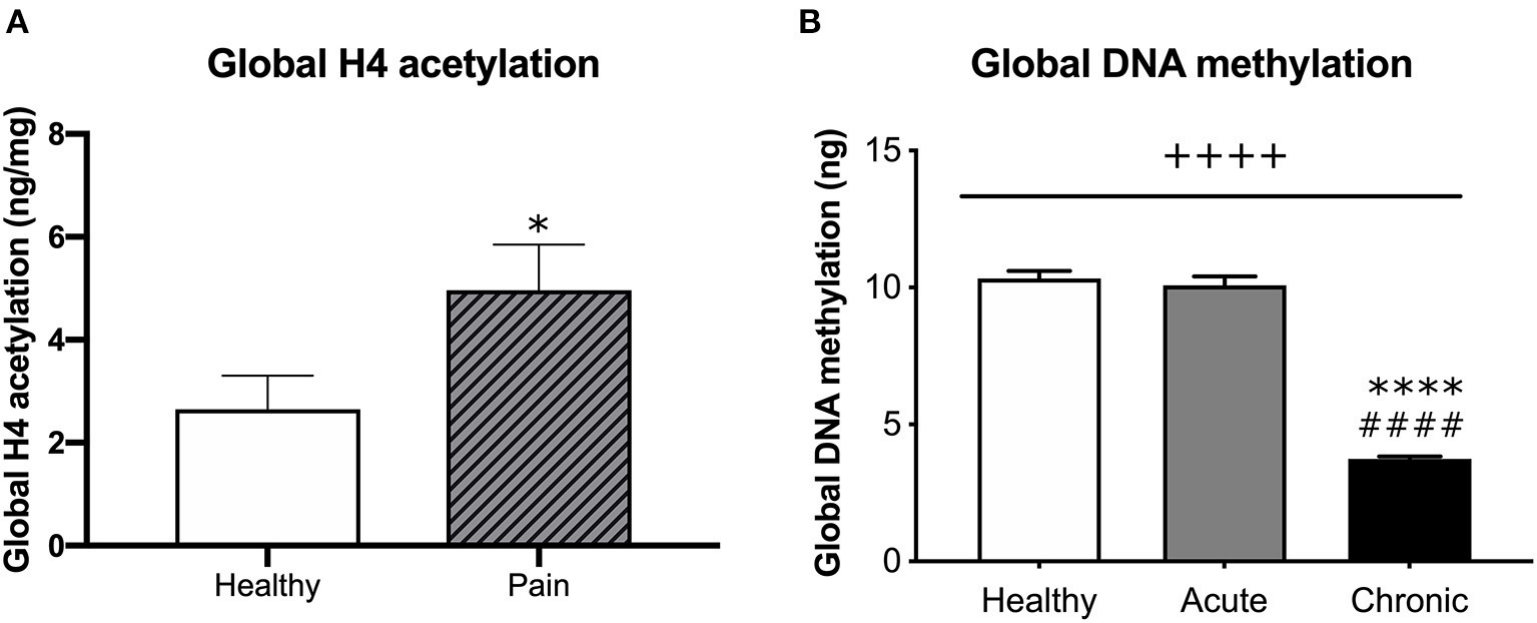
**(A)** Comparison of global H4 acetylation between healthy participants and participants that presented with pain (aLBP and cLBP). Data presented as H4 acetylation (ng/mg of total protein) + SEM. ^*^p < 0.05 vs. healthy controls. **(B)** Global DNA methylation was quantified from healthy controls, aLBP, and cLBP participants. Data presented as mean ng methylation per 150 ng total DNA + SEM. ++++*p* < 0.000 group effect; ^****^p < 0.0000 vs. healthy controls, ^####^*p* < 0.0000 vs. aLBP.

We measured the level of global histone H4 acetylation in the histone extract from blood taken at the first visit. Although we did not observe a significant difference in histone H4 acetylation level in aLBP vs. cLBP participants (*p* > 0.05), when both pain groups were combined, we observed a significantly higher amount of global histone H4 acetylation compared to healthy controls (*p* < 0.05, *t* = 2.261; Figure 3A), suggesting a relationship between the experience of pain and histone acetylation that is likely independent of pain chronicity.

### Correlation of QST With H4 Acetylation

We assessed the potential relationship between H4 acetylation at time of pain onset and QST measurements. We found that MPS, WUR1, and WDT at the site of pain were positively correlated with H4 acetylation (all r_p_ > 0.325, *p* < 0.05; Table 5). Conversely, CDT at the site of pain was negatively correlated with H4 acetylation (r_p_ = −0.315, *p* < 0.05). At the control site, WUR10 and WDT were positively correlated with H4 acetylation (r_p_> 0.297, *p* < 0.05) and MPT and CDT were negatively correlated with H4 acetylation (r_p_ > −0.312, *p* < 0.05).

**Table 5:** Correlation of quantitative sensory testing (QST) measures and H4 acetylation at the site of pain and the control site.

**QST measurement and site**	**Correlation with H4 acetylation at pain onset**	***p-*** **value**
Pain pressure threshold		
Low back	0.044	*p =* 0.777
Control site	0.093	*p =* 0.545
Mechanical detection threshold		
Low back	0.058	*p =* 0.704
Control site	0.204	*p =* 0.178
Mechanical pain threshold		
Low back	−0.245	*p =* 0.105
Control site^*^	–**0.312**	***p*** **= 0.037**
Mechanical pain sensitivity		
Low back^*^	**0.325**	***p*** **= 0.029**
Control site	0.154	*p =* 0.313
Dynamic mechanical allodynia		
Low back	0.221	*p =* 0.144
Control site	0.252	*p =* 0.095
Wind up ratio		
Low back measurement 1^*^	**0.325**	***p*** **= 0.029**
Low back measurement 10	0.136	*p =* 0.374
Wind up ratio		
Control site measurement 1	0.193	*p =* 0.205
Control site measurement 10^*^	**0.297**	***p*** **= 0.047**
Vibration detection threshold		
Low back	0.191	*p =* 0.208
Control site	0.042	*p =* 0.782
Cold detection threshold		
Low back^*^	**−0.315**	***p*** **= 0.035**
Control site^*^	**−0.520**	***p*** **= 0.0002**
Cold pain threshold		
Low back	0.136	*p =* 0.371
Control site	**–**0.021	*p =* 0.892
Warm detection threshold		
Low back^*^	**0.408**	***p*** **= 0.005**
Control site^*^	**0.384**	***p*** **= 0.009**
Heat pain threshold		
Low back	0.109	*p =* 0.477
Control site	0.150	*p =* 0.324

* *p < 0.05*.

### Global DNA Methylation in cLBP Participants Is Significantly Less Than aLBP Participants and Healthy Controls

Whole blood was collected from each participant by venipuncture at the time of the first study visit. DNA (150 ng) was then isolated from blood samples and global DNA methylation was quantified for each participant. We found a significant group effect on global gDNA methylation levels [*F*_(2, 41)_ = 213.40, *p* < 0.05; Figure 3B]. *Post-hoc* analyses revealed that cLBP participants had significantly lower global DNA methylation relative to aLBP participants and healthy controls (*p* < 0.05). Methylation levels did not differ between aLBP and healthy controls (*p* > 0.05).

### Global DNA Methylation Correlation With mRNA Expression of Candidate Genes

**Figure 4:**
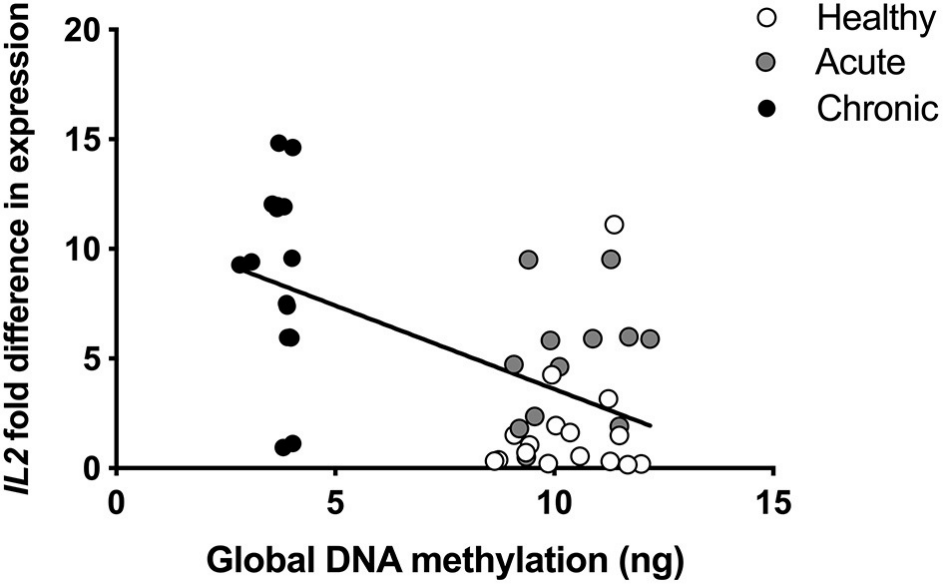
Pearson correlation of global DNA methylation and interleukin 2 (*IL2)* expression in blood collected at the time of pain onset. r_*p*_= −0.569.

mRNA was also isolated from participant blood and analyzed using an array of 84 pain-relevant genes. Expression levels were then correlated with global DNA methylation levels to determine the relationship between global methylation and pain-related gene expression. Expression levels of brain derived neurotrophic factor (*BDNF*), Cx3C motif chemokine receptor 1 (*CX3CR1*), GTP cyclohydrolase 1 (*GCH1*), purinergic receptors, (*P2RX4, P2RX7*, and *P2RY1)*, prostaglandin E synthase 3 (*PTGES3*), and tumor necrosis factor (*TNF*) were positively correlated with global DNA methylation levels (all r_p_ > 0.481; Table 6). Interestingly, expression of Interleukin 2 (*IL2*) was the only gene we found to be negatively correlated with global DNA methylation (r_p_ = −0.569; Figure 4) at both timepoints in the cLBP condition.

**Table 6:** Significant correlations of global DNA methylation with mRNA expression of pain-related genes.

**Gene**	**Correlation with global DNA methylation at pain onset**	***p*** **-value**	**cLBP 6 month visit correlation with global DNA methylation at pain onset**	***p*** **-value**
*BDNF*	0.491	*p =* 0.0003		
*CX3CR1*	0.526	*p =* 0.0001		
*GCH1*	0.482	*p =* 0.0004		
*IL2*	−0.569	*p =* 0.00003	−0.814	*p =* 0.00006
*P2RX4*	0.589	*p =* 0.00001	−0.641	*p =* 0.007
*P2RX7*	0.487	*p =* 0.0004		
*P2RY1*	0.495	*p =* 0.0003		
*PTGES3*	0.495	*p =* 0.0003		
*TNF*	0.481	*p =* 0.0005		

*BDNF, brain derived neurotrophic factor; CX3CR1, Cx3C motif chemokine receptor 1; GCH1, GTP cyclohydrolase 1; P2RX4, P2RX7, and P2RY1, purinergic receptors 4, 7, and 1; PTGES3, prostaglandin E synthase 3; TNF, tumor necrosis factor*.

Pearson correlations were also conducted to determine whether global DNA methylation taken at the start of the study correlated with gene expression level at the 6-month time point. As in the initial analysis, *IL2* expression was significantly negatively correlated (r_p_ = −0.814) with DNA methylation at the time of study initiation (*p* < 0.05; Table 6) as was P2RX4 (r_p_ = −0.641, *p* < 0.05).

### cLBP Participants Have Significantly Greater *IL2* mRNA Expression Than aLBP Participants and Healthy Controls

**Figure 5:**
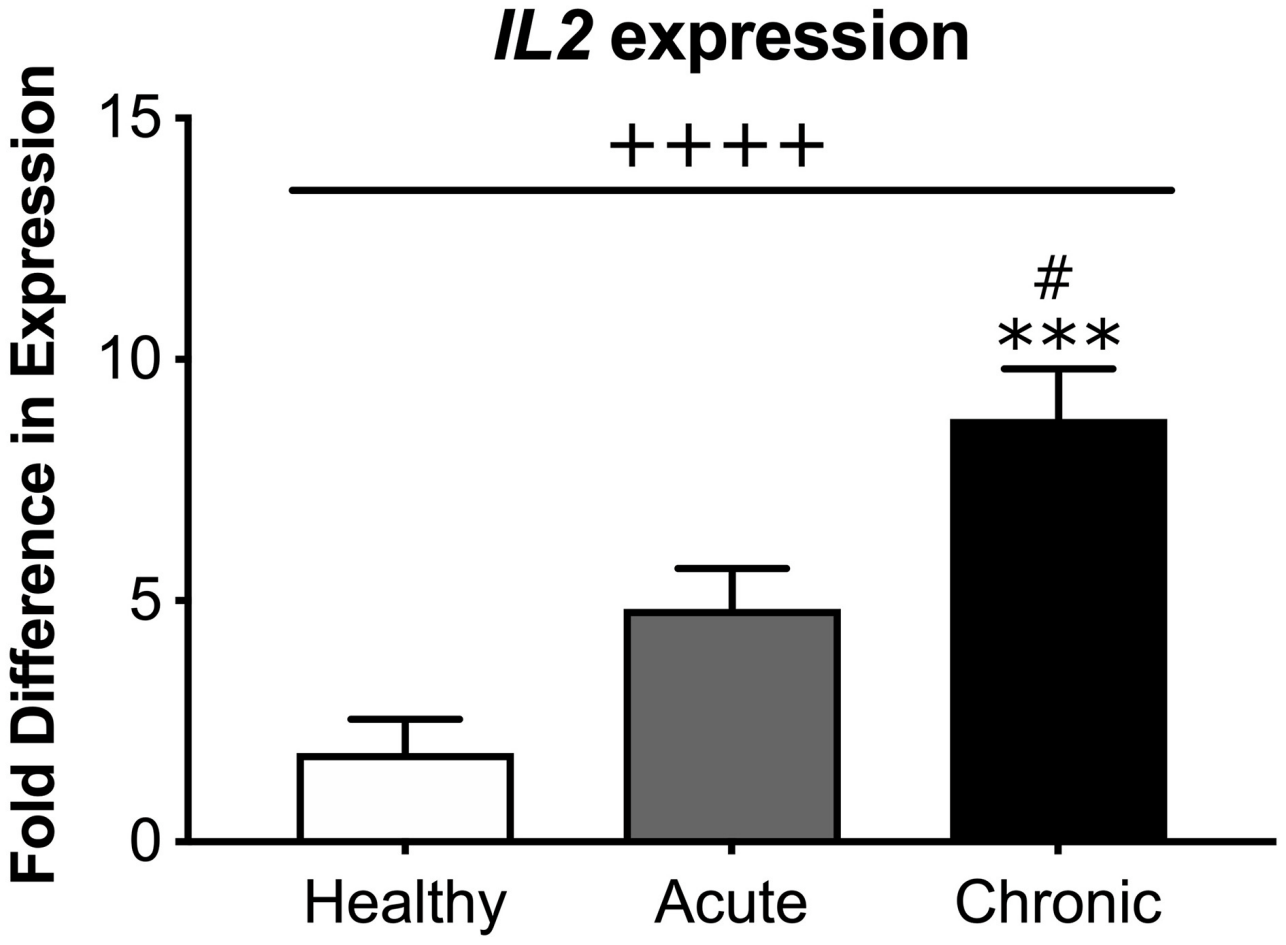
Systemic interleukin 2 (*IL2)* mRNA expression. There was an overall effect of group on *IL2* expression measured from blood samples of participants. cLBP participants had significantly greater fold difference in *IL2* expression compared to aLBP participants and healthy controls. ++++*p* < 0.0000 group effect, ^***^p < 0.0001 vs. healthy controls, ^#^*p* < 0.05 vs. aLBP.

Because we found a significant inverse relationship between global DNA methylation and *IL2* mRNA expression, we then directly compared levels of *IL2* mRNA expression between each participant condition. Our analysis revealed that cLBP participants had significantly greater *IL2* mRNA expression relative to aLBP participants and healthy controls [*F*_(2, 41)_ = 16.937, *p* < 0.05; Figure 5].

### Correlation of QST With Global DNA Methylation

Next, we explored the relationship between global DNA methylation and QST measurements. We found that MPS, ALL, WUR1, WUR10, and WDT at the site of pain were negatively correlated with global DNA methylation (all r_p_ > −0.407, *p* < 0.05; Table 7). Conversely, MPT at the site of pain was positively correlated with global DNA methylation (r_p_ = 0.302, *p* < 0.05). Our analysis also revealed that MPS, ALL, WUR1, and WUR10 at the control site were negatively correlated with global DNA methylation (all r_p_ > −0.314, *p* < 0.05).

**Table 7:** Correlation of quantitative sensory testing (QST) at the site of pain and the control site with methylation.

**QST measurement and site**	**Correlation with global DNA methylation at pain onset**	***p*** **-value**
Pain pressure threshold		
Low back	0.210	*p =* 0.166
Control site	0.053	*p =* 0.731
Mechanical detection threshold		
Low back	0.028	*p =* 0.854
Control site	−0.209	*p =* 0.168
Mechanical pain threshold		
Low back^*^	**0.302**	***p*** **= 0.044**
Control site	0.125	*p =* 0.412
Mechanical pain sensitivity		
Low back^*^	−**0.505**	***p*** **= 0.0004**
Control site^*^	−**0.348**	***p*** **= 0.019**
Dynamic mechanical allodynia		
Low back^*^	−**0.521**	***p*** **= 0.0002**
Control site^*^	−**0.314**	***p*** **= 0.035**
Wind up ratio		
Low back measurement 1^*^	−**0.505**	***p*** **= 0.0004**
Low back measurement 10^*^	−**0.408**	***p*** **= 0.005**
Wind up ratio		
Control site measurement 1^*^	−**0.389**	***p*** **= 0.008**
Control site measurement 10^*^	−**0.432**	***p*** **= 0.003**
Vibration detection threshold		
Low back	0.286	*p =* 0.056
Control site	−0.082	*p =* 0.591
Cold detection threshold		
Low back	0.285	*p =* 0.057
Control site	0.216	*p =* 0.154
Cold pain threshold		
Low back	−0.241	*p =* 0.111
Control site	−0.115	*p =* 0.451
Warm detection threshold		
Low back^*^	−**0.443**	***p*** **= 0.002**
Control site	−0.193	*p =* 0.204
Heat pain threshold		
Low back	−0.126	*p =* 0.408
Control site	−0.110	*p =* 0.473

* *p < 0.05*.

### Correlation of BPI With Global DNA Methylation

We also correlated global DNA methylation with BPI self-reports of pain, and found that BPI worst, BPI least, BPI average, BPI now, and BPI interference were all negatively correlated with global DNA methylation (all r_p_ > −0.379, *p* < 0.05; Table 8). As expected, no relationship was found between BPI relief and global DNA methylation (*p* = 0.428).

**Table 8:** Brief pain inventory (BPI) subscales correlation with DNA methylation.

**BPI subscale**	**Correlation with global DNA methylation at pain onset**	***p*** **-value**
BPI worst	−0.513^*^	***p*** **= 0.005**
BPI least	−0.533^*^	***p*** **= 0.003**
BPI average	−0.380^*^	***p*** **= 0.046**
BPI now	−0.546^*^	***p*** **= 0.003**
BPI relief	−0.159	*p =* 0.428
BPI interference	−0.443^*^	***p*** **= 0.018**

* *p < 0.05*.

### Correlation of QST With *IL2*

We found that the only gene whose expression was significantly negatively correlated with global DNA methylation in cLBP participants was *IL2*. Therefore, we examined whether any QST measurements were also related to *IL2* mRNA expression levels and found that only cold pain threshold (CPT) at the site of pain was positively correlated with *IL2* expression (r_*p*_ = 0.390, *p* < 0.05).

### Linear Regression of QST With Methylation and *IL2*

We used multiple stepwise linear regression to further study the relationship between QST measurements, global DNA methylation, and *IL2* expression. These analyses revealed that MPT, MPS, ALL, WUR1, WUR10, and WDT at the site of pain were all associated with global DNA methylation, all *F*s > 4.3, *r*^2^ > 0.097, and *p* < 0.05 (Table 9). Additionally, MPS, ALL, WUR1, and WUR10 at the control site were also associated with global DNA methylation all *F*s>4.2, all *r*^2^ > 0.096, *p* < 0.05. When *IL2* was added to the model, we found that inclusion of *IL2* did not account for any additional variance. Interestingly, it was only when both methylation and *IL2* were added to the model that MPT at the control site that this relationship reached statistical significance, *F*_(1, 39)_ = 4.885, *r*^2^= 0.127, *p* < 0.05.

**Table 9:** Stepwise linear regression of quantitative sensory testing QST with methylation (step 1) and *IL2* (step 2).

**QST measurement and site**	**Model**	***r*** **^2^**	**F change**	***p*** **-value**
Pain pressure threshold_ low back	1	0.047	1.993	*p =* 0.166
	2	0.052	0.171	*p =* 0.681
Pain pressure threshold_ control site	1	0.001	0.051	*p =* 0.823
	2	0.056	2.255	*p =* 0.141
Mechanical detection threshold _ low back	1	0.001	0.053	*p =* 0.820
	2	0.036	1.399	*p =* 0.244
Mechanical detection threshold_ control site	1	0.050	2.122	*p =* 0.153
	2	0.087	1.553	*p =* 0.220
Mechanical pain threshold_ low back	1^*^	**0.097**	**4.299**	***p*** **= 0.045**
	2	0.119	0.957	*p =* 0.334
Mechanical pain threshold_ control site	1	0.018	0.736	*p = 0*.396
	2^*^	**0.127**	**4.885**	***p*** **= 0.033**
Mechanical pain sensitivity_ low back	1^*^	**0.276**	**15.242**	***p*** **= 0.0003**
	2	0.290	0.797	*p =* 0.378
Mechanical pain sensitivity_ control site	1^*^	**0.123**	**5.622**	***p*** **= 0.023**
	2	0.129	0.257	*p =* 0.615
Dynamic mechanical allodynia_ low back	1^*^	**0.276**	**15.246**	***p*** **= 0.0003**
	2	0.283	0.383	*p =* 0.539
Dynamic mechanical allodynia_ control site	1^*^	**0.096**	**4.243**	***p*** **= 0.046**
	2	0.102	0.285	*p =* 0.597
Wind up ratio_ low back measurement 1	**1**	**0.276**	**15.242**	***p*** **= 0.0003**
	2	0.290	0.797	*p =* 0.378
Wind up ratio_ low back measurement 10	1^*^	**0.161**	**7.656**	***p*** **= 0.009**
	2	0.162	0.080	*p =* 0.779
Wind up ratio_ control site measurement 1	1^*^	**0.155**	**7.309**	***p*** **= 0.010**
	2	0.186	1.494	*p =* 0.229
Wind up ratio_ control site measurement 10	1^*^	**0.178**	**8.668**	***p*** **= 0.005**
	2	0.229	2.597	*p =* 0.115
Vibration detection threshold _ low back	1	0.083	3.597	*p =* 0.065
	2	0.037	0.068	*p =* 0.795
Vibration detection threshold_ control site	—+	—+	—+	—+
Cold detection threshold_ low back	1	0.077	3.317	*p =* 0.076
	2	0.078	0.081	*p =* 0.777
Cold detection threshold_ control site	1	0.051	2.163	*p =* 0.149
	2	0.067	0.645	*p =* 0.427
Cold pain threshold_ low back	1	0.071	3.047	*p =* 0.089
	2	0.074	0.131	*p =* 0.719
Cold pain threshold_ control site	1	0.019	0.759	*p =* 0.389
	2	0.026	0.298	*p =* 0.588
Warm detection threshold_ low back	1^*^	**0.196**	**9.769**	***p*** **= 0.003**
	2	0.230	1.701	*p =* 0.200
Warm detection threshold_ control site	1	0.041	1.693	*p =* 0.201
	2	0.072	1.311	*p =* 0.259
Heat pain threshold_ low back	1	0.014	0.551	*p =* 0.462
	2	0.016	0.095	*p =* 0.760
Heat pain threshold_ control site	1	0.010	0.394	*p =* 0.534
	2	0.013	0.143	*p =* 0.708

* *p < 0.05. + control site vibration detection threshold (VDT) was the minimum score for all participants at both time points, so correlation was not calculated*.

### Linear Regression of BPI With Methylation and *IL2*

Finally, we examined the relationship between BPI subscales, DNA methylation, and *IL2* expression using multiple stepwise linear regression. Our analysis revealed that BPI worst, BPI least, BPI average, BPI now, and BPI interference were significantly associated with DNA methylation (all Fs >5.05, *r*^2^ > 0.174, and *p* < 0.05; Table 10). However, adding *IL2* did not increase the amount of variance accounted for by the model, suggesting that the pain effects of IL2 are explained by the methylation status of the participants.

**Table 10:** Stepwise linear regression of Brief Pain Inventory (BPI) with methylation (step 1) and *IL2* (step 2).

**BPI subscale**	**Step**	***R*** **^2^**	**F change**	***p*** **-value**
BPI worst	1^*^	**0.319**	**11.243**	***p*** **= 0.003**
	2	0.322	0.094	*p =* 0.762
BPI least	1^*^	**0.296**	**10.075**	***p*** **= 0.004**
	2	0.347	1.821	*p =* 0.190
BPI average	1^*^	**0.174**	**5.055**	***p*** **= 0.034**
	2	0.178	0.118	*p =* 0.734
BPI now	1^*^	**0.338**	**12.248**	***p*** **= 0.002**
	2	0.366	1.022	*p =* 0.323
BPI relief	1	0.058	1.358	*p =* 0.256
	2	0.096	0.879	*p =* 0.359
BPI interference	1^*^	**0.216**	**6.601**	***p*** **= 0.017**
	2	0.224	0.257	*p =* 0.617

* *p < 0.05*.

## Discussion

Low back pain is one of the most common pain conditions, affecting nearly 1 in 10 people worldwide (1). Because not all individuals will transition from aLBP to cLBP, it is imperative to determine factors with predictive potential for transitioning to cLBP in order to identify those at the highest risk and develop precision pain medicine interventions. The present study was designed to examine whether differences in epigenetic modifications (global DNA methylation and/or H4 histone acetylation status), candidate gene expression, and/or somatosensory functioning (QST) could distinguish participants whose pain would resolve quickly (aLBP) from those who would eventually transition to cLBP.

Participants who would later develop cLBP exhibited decreased global DNA methylation status compared to participants whose pain resolved quickly (aLBP) and healthy controls, indicating a potential role for hypomethylation in subsequent candidate gene expression changes contributing to pain chronicity. We further found that a single candidate gene, *IL2*, was negatively correlated with global DNA methylation at both pain onset and 6 months later, a time when participants had transitioned to cLBP. In addition, multiple measures of somatosensory function were also associated with global DNA methylation which may reflect the contribution of epigenetic modifications to fundamental sensory processing changes seen in cLBP. While the present analyses do not test a hypothesized mechanism underlying the transition from aLBP to cLBP, our findings shed light on potential novel hypotheses for subsequent testing in other larger cohorts.

Histone acetylation occurs on the n-terminal of the histone tail and makes chromatin more accessible for transcription factors to bind (23), which usually leads to an increase in transcription (24, 25). Altered histone acetylation status has been associated with both inflammatory and neuropathic pain (26) in preclinical models. Interestingly, inhibitors of histone acetyltransferases, which add acetyl groups, and histone deacetylases, which remove the acetyl groups have been shown to relieve pain (35, 36) albeit through different mechanisms (26). We found no differences in H4 acetylation status between aLBP and cLBP at the time of pain onset although when both groups were combined, they were found to have elevated H4 acetylation compared to healthy controls. This pattern suggests that H4 acetylation may contribute to or result from the presence of acute pain but does not appear to discriminate those at increased risk for transition to chronic pain. As a result, we prioritized the examination of potential relationships between global DNA methylation, candidate gene expression, somatosensory function (QST) and pain phenotype (chronicity).

cLBP participants had significantly lower global DNA methylation compared to aLBP and healthy controls, in line with other studies that report differences in DNA methylation patterns in individuals with chronic pain (14–16, 18, 37), including cLBP (19). For example, Aroke et al., found that individuals with non-specific cLBP had 159 differentially methylated regions compared to healthy controls, the majority of which were located in CpG islands and promoter regions (19) for genes involved in immune signaling, endochondral ossification, and G-protein coupled transmissions. DNA methylation has also been implicated in the etiology of fibromyalgia (FM) (14, 18). Ciampi de Andrade et al., found 1,610 differentially methylated DNA positions in FM patients, the vast majority (69%) of which were hypomethylated (18). Genes associated with these CpG sites included those involved in metabolic pathways, the mitogen-activated protein kinase signaling pathway, and regulatory pathways of the actin cytoskeleton, among others. Conversely, Menzies et al., found 69 differentially methylated sites in a cohort of FM patients with most being hypermethylated compared to healthy controls (14) and an overrepresentation of genes involved in neuron differentiation and nervous system development. In a cohort of patients with limb amputations, the subset that developed complex regional pain syndrome (CRPS) had 48 differentially methylated CpG sites compared to individuals that developed non-CRPS neuropathic pain (37) with all but 7 of these sites found to be hypomethylated. Finally, a study assessing whole genome DNA methylation found significant differences in DNA methylation profiles in individuals with early vs. advanced intervertebral disc degeneration (38). Four loci were hypomethylated while 216 were hypermethylated in the advanced disc degeneration stage but none of these alterations in methylation were assessed for their association with pain, *per se*.

Hypomethylation is most often associated with increased gene expression (13), so we identified a single gene from our quantified 84 pain-related candidate genes, *IL2*, whose expression was significantly *negatively* correlated with DNA methylation level at both the time of pain onset and after transition to chronic pain. Eight other candidate genes were significantly *positively* correlated with global DNA methylation status at the time of pain onset: *BDNF, CX3CR1, GCH1, P2RX4, P2RX7, P2RY1, PTGES3, and TNF*. Expression levels of each of these candidate genes have previously been associated with various chronic pain conditions in humans and preclinical models (39–46), but these *positively* correlated candidate genes are unlikely to reflect the impact the hypomethylation noted in the cLBP group. As a result, *IL2* was identified as the highest priority candidate gene.

cLBP participants exhibited nearly a 5-fold increase in *IL2* expression relative to aLBP participants at pain onset. The *IL2* gene encodes a cytokine that has been shown in preclinical studies to have both algesic (47, 48) and analgesic properties (49, 50). This conflict in the literature may depend on the pain model, location of *IL2* gene or IL2 protein manipulation, as well as the pain-like behavior measured but points to the role of *IL2* as a potent pain modulator. For example, studies examining thermal sensitivity after chronic constriction injury found that intrathecal administration of human *IL2* gene transfection resulted in analgesia (49, 50), whereas others examining mechanical hypersensitivity following an intraplantar (47) or intraarticular (48) injection of IL2 protein found it to be algesic. These data suggest that an increased level of peripheral IL2, in particular, may contribute to the transition from aLBP to cLBP. This result suggests that expression of *IL2* is elevated early in individuals with pain and is maintained in those individuals that eventually transition to a cLBP state.

*IL2* has been shown to exert both anti- or pro-inflammatory (51) influence, but our data support the pro-inflammatory interpretation as *IL2* remained increased in the cLBP group over time. Prior reports indicate patients with painful neuropathy exhibit 2-fold higher *IL2* mRNA expression in circulation compared to healthy controls and 2-fold higher *IL2* mRNA and IL2 protein compared to patients with painless neuropathy (52). Similarly, *IL2* expression is higher in the blood of individuals with CRPS compared to healthy controls (53). We hypothesize that cells circulating in the blood, such as immune cells, produce IL2 due to decreased methylation of the *IL2* gene. Taken together with prior reports, our data point to an algesic role for IL2 through its role as a proinflammatory mediator that fails to resolve in individuals with cLBP potentially resulting in peripheral nociceptor sensitization and persistent enhanced pain perception. Further work is needed to test this, and other, specific mechanisms in the transition to chronic pain.

*P2RX4* is a gene that encodes the P2RX4 protein, which belongs to the family of receptors for ATP. In our participants, the expression level of *P2RX4* went from a positive correlation with global DNA methylation at pain onset to a negative correlation at the 6 month time point, which suggests that increased *P2RX4* signaling may play a separate role in the initiation and maintenance cLBP and that only the long term effects are under the influence of DNA methylation. This is in agreement with other studies that implicate *P2RX4* in the chronification of neuropathic pain (54), potentially through astrocyte or other glial cell activation (55, 56). Interestingly, *P2RX4* and *IL2* appear to be linked as inhibition of *P2RX4* has been shown to inhibit *IL2* transcription (57) and ATP is required for *IL2* transcription in immune cells (58). This represents one mechanism implicating both *P2RX4* and *IL2* in early balance of inflammatory responding and risk for pain chronicity.

In agreement with our previous reports (6, 27, 28) that included a larger cohort of these participants, cLBP participants displayed higher BPI subscale scores compared to aLBP participants at pain onset. cLBP participants also displayed altered somatosensory function across multiple domains measured at the site of pain compared to aLBP participants. Moreover, for the cLBP participants, multiple somatosensory function (QST) measures were correlated over time between pain onset and the 6 month follow up visit, indicating that increased sensitivity at the time of recruitment corresponded to a persistent increase in sensitivity over time. Only dynamic mechanical allodynia (ALL) taken at the control site which was found to increase over time for those in the cLBP group, suggesting that the mechanisms underlying ALL are engaged early on and escalate over time. Several QST measurements were negatively correlated with global DNA methylation as were all of the BPI subscales, with the exception of BPI relief. When we examined the relationship of *IL2* expression with the QST measures, CPT at the site of pain was the only variable significantly associated with *IL2* expression, indicating that increased cold sensitivity may be modulated by *IL2* expression. Cold pain sensitivity has been shown elsewhere to be a predictive factor in the development of long-term pain (59) and others have reported a positive correlation between IL2 plasma concentrations and increasing pain intensity in chronic pain patients (60). While we prioritized potential associations with IL2, other pain-related candidate genes could also influence somatosensory function and/or chronic pain development.

Further evaluation of the associations of QST measures and BPI with methylation and *IL2* expression revealed that methylation status, not *IL2* expression level, was a major predictor of QST outcomes as adding *IL2* to the model did not account for any additional variance in the regression analysis, suggesting the methylation effects and *IL2* expression reflect a common process. Bruniquel and Schwartz describe a close relationship between *Il2* expression and DNA methylation status in preclinical mouse models (61). They found both *in vitro* and *in vivo* evidence indicating that naïve T lymphocytes are highly methylated in the promoter-enhancer region of *Il2*, and therefore do not express *Il2*. Conversely, when T cells are activated, many of the CpG sites in the *Il2* promoter-enhancer region are demethylated and *Il2* is expressed. Clearly, active demethylation of the *IL2* promoter region plays a key role in its expression, however the mechanism behind this remains unclear (62).

Our data align with previous studies demonstrating that individuals that will develop cLBP display significant differences in somatosensory function at pain onset compared to individuals whose pain will resolve. Further, our data suggest that global DNA methylation status is an epigenetic predictor of individuals that eventually transition from aLBP to cLBP, while histone acetylation is more accurately linked to an active pain state than to the risk for chronic pain transition. Finally, *IL2* significantly negatively correlated with DNA methylation level and was significantly higher in our cLBP participants even at the onset of the low back pain episode. This research is significant because while many interventions for the treatment of cLBP exist, few are universally successful in reducing pain and increasing quality of life (63) once pain is established. This type of profiling has already been recognized as a way to identify subgroups of neuropathic pain patients and to personalize their pain management program (64). Improving prediction of those who will transition to cLBP will allow us to intervene before the transition to the more treatment-resistant chronic pain condition.

The current study has several caveats including the relatively small sample size and the use of global epigenetic modification data with specific candidate gene expression. While genotyping studies require substantial sample sizes, the use of candidate gene expression and quantifiable epigenetic measures are more amenable to analysis in smaller study cohorts. That being said, we did have a significantly greater percentage of Black individuals in our cLBP group, but we did not control for race in our analyses due to the small sample size and limited variance. However, previous studies have shown that Black individuals experience more frequent and severe chronic-pain compared to non-Hispanic whites (65), including back pain (66). Interestingly, this has been suggested to be due, in part, to epigenetic changes associated with increased adversity, stress, and racial discrimination (67), our findings support this theory but do not test it directly. Future studies in larger cohorts are warranted to tease apart the interrelationships between race, epigenetic status, gene expression, and chronic pain. An additional limitation is that we measured a subset of global epigenetic changes in our participants (i.e., global H4 acetylation and global DNA methylation). There are other histone modifications and gene specific epigenetic modifications that are not assessed with these methods. We acknowledge that more specific measurements could be taken, such as acetylation at specific lysine sites or methylation of specific gene promoters, including those for *IL2*, should be addressed in future work.

## Data Availability Statement

The original contributions presented in the study are included in the article, further inquiries can be directed to the corresponding author.

## Ethics Statement

The studies involving human participants were reviewed and approved by University of Connecticut Institutional Review Board. The patients/participants provided their written informed consent to participate in this study.

## Author Contributions

OCE: data collection, data analyses, and manuscript preparation. NG, BK, NM, MP, and KMBC: data collection and data analyses. AS, EEY, and KMB: study design, supervision of data collection, and manuscript preparation. All authors contributed to the article and approved the submitted version.

## Funding

This work was supported by R03NS096454-02, R21NS104789, Rita Allen Foundation Award in Pain (to KMB), R01 NR013932 (to AS), and NINR T32 NR013456 (mPIs) (to KMBC).

## Conflict of Interest

The authors declare that the research was conducted in the absence of any commercial or financial relationships that could be construed as a potential conflict of interest.

## Publisher's Note

All claims expressed in this article are solely those of the authors and do not necessarily represent those of their affiliated organizations, or those of the publisher, the editors and the reviewers. Any product that may be evaluated in this article, or claim that may be made by its manufacturer, is not guaranteed or endorsed by the publisher.
